# SiDCoN: A Tool to Aid Scoring of DNA Copy Number Changes in SNP Chip Data

**DOI:** 10.1371/journal.pone.0001093

**Published:** 2007-10-31

**Authors:** Derek J. Nancarrow, Herlina Y. Handoko, Mitchell S. Stark, David C. Whiteman, Nicholas K. Hayward

**Affiliations:** 1 Oncogenomics, Queensland Institute of Medical Research, Herston, Queensland, Australia; 2 Cancer and Population Studies, Queensland Institute of Medical Research, Herston, Queensland, Australia; University of Minnesota, United States of America

## Abstract

The recent application of genome-wide, single nucleotide polymorphism (SNP) microarrays to investigate DNA copy number aberrations in cancer has provided unparalleled sensitivity for identifying genomic changes. In some instances the complexity of these changes makes them difficult to interpret, particularly when tumour samples are contaminated with normal (stromal) tissue. Current automated scoring algorithms require considerable manual data checking and correction, especially when assessing uncultured tumour specimens. To address these limitations we have developed a visual tool to aid in the analysis of DNA copy number data. Simulated DNA Copy Number (SiDCoN) is a spreadsheet-based application designed to simulate the appearance of B-allele and logR plots for all known types of tumour DNA copy number changes, in the presence or absence of stromal contamination. The system allows the user to determine the level of stromal contamination, as well as specify up to 3 different DNA copy number aberrations for up to 5000 data points (representing individual SNPs). This allows users great flexibility to assess simple or complex DNA copy number combinations. We demonstrate how this utility can be used to estimate the level of stromal contamination within tumour samples and its application in deciphering the complex heterogeneous copy number changes we have observed in a series of tumours. We believe this tool will prove useful to others working in the area, both as a training tool, and to aid in the interpretation of complex copy number changes.

## Introduction

Single nucleotide polymorphism (SNP) microarrays provide data on both genotype and signal intensity, the combination of which can be used to generate information on chromosomal segment copy number. An increasing number of studies utilise whole-genome high density SNP chips to generate DNA copy number profiles for a variety of tumour types. Kits and software tools are now commercially available for this purpose from a number of suppliers. This emerging technology has distinct advantages over previous karyotype-based comparative genome hybridization (CGH) methods [1] and analytic methods are evolving rapidly. When applying these SNP microarrays (SNP-aCGH) to cancer research, the aim is to synthesize a comprehensive DNA copy number profile which maps aberrations across the entire genome within individual tumour samples.

There are several method papers devoted to the analysis of DNA copy number using SNP array platforms [2]–[5] and dedicated software functions are available in commercial applications. There are two broad approaches to this work: 1) identifying statistically significant genomic regions of change (e.g. Colella and coworkers [2]); 2) developing tools to auto-analyse the data to generate genome-wide, sample specific DNA copy number profiles.

The success of SNP-aCGH for mapping sample specific DNA copy number changes stems from the ability to combine CGH and loss of heterozygosity (LOH) studies in the same analysis. As is often the case with new biotechnology, the analysis procedures lag behind the experimental advancements in terms of simplicity and flexibility. While commercially available software applications provide analysis algorithms to identify significant regions of change, we [6] have found this to be inadequate for generating a whole-genome view of DNA copy number changes without heavy manual interpretation.

In SNP-aCGH analyses the resulting genotype data consist of intensity values for two channels corresponding to the fluorophors associated with the A & B alleles (attached to specific oligos/beads). Data can be plotted as raw A versus raw B intensity plots, however several refined data presentation methods have proven more useful. One of these, log 2 of the sample intensity to reference intensity ratio (logR), provides a continuous measure of the CGH component of the data. In this case, the signal intensity of each SNP in the target sample is expressed as a ratio over that of the normal sample or reference pool. Log 2 of this ratio provides an effective means to curtail the range of outlying values. While the variability of individual logR values is large, due to variances in PCR conditions and primer sequences, modified algorithms such as that of Nannya and coworkers [7] and the Illumina proprietary method, as well as the application of a moving average, are available to reduce the effects of this variation across a chromosomal region. These features, including a proprietary algorithm for SNP normalisation, are built in to the Illumina Beadstudio 2&3 software packages.

Another key SNP-aCGH data presentation track, Allele B frequency (Ballele), visualises the LOH component. By adjusting the theta (allelic intensity ratio) value for cluster position (assessed in a panel of normal samples) the relative composition of each SNP allele can be determined [4]. The Allele B frequency represents the proportion contributed by one SNP allele (B), expressed as a range from 0 to 1. Thus in a normal heterozygous sample (with equal amounts of allele A and allele B for a given SNP) Ballele would be 0.5. Homozygosity is represented by either 0 (AA) or 1 (BB).

As with traditional LOH analysis by microsatellite or SNP markers, a run of consecutive homozygous markers indicates a region of potential DNA loss or copy neutral LOH. Intermediate values (>0 & <0.5 or >0.5 & <1) represent variation in the amount of allele B relative to the total (A+B) SNP intensity. This can be seen, for example, as a result of incomplete loss of one allele. Ballele plots tend to be less variable than those of logR [4]. The methods for calculating both Ballele and logR are described in detail by Peiffer and coworkers [4].

When considered together, Ballele and logR plots of SNP-aCGH data allow the relative amounts of each parental marker to be estimated across each chromosome. It is unnecessary to type parents for SNP-aCGH, thus the maternal or paternal origin of an individual chromosome is unknown. However, as one delves into deeper levels of DNA copy number complexity there is a need to be able to clearly express which genotype is being referred to. As an example, a 3n genotype (simple amplification) can consist of chromosome segments from the same parent (*AAA*) or an imbalance of chromosomes from both parents (*AAB, ABB*). SNP-aCGH can distinguish *AAA* from the other two forms of 3n amplification (which would require parental information to be separated), and there may be a biological distinction; does a target gene within an *AAA* region function in the context of tumour suppression, or as an oncogene? In this manuscript we have designated *A* and *B* to distinguish between paired chromosomes to allow a more detailed description of the observable genotypes. Note that italics have been used to distinguish *A* & *B* parental chromosomes from the A and B alleles at an individual SNP. We chose this nomenclature since “A” and “B” have historically been used extensively in both instances.

Interpreting Ballele and logR plots together provides an estimate of the average copy number of *A* and *B* versions of each autosome, thus providing a sample specific genome-wide profile of DNA copy number. These views essentially correspond to data generated from the original CGH (∼logR) and LOH (∼Ballele) methodologies and it is the ability to simultaneously interpret both in a very dense map that makes SNP-aCGH so powerful.

When compared to cancer cell lines, DNA from a tumour biopsy often generates a distinctively different pattern (Ballele & logR) for a variety of the common copy number changes observed when using SNP microarrays. This is due to the presence of non-cancer cells within the biopsy, including inflammatory cells, connective tissues and other components referred to as stromal contamination (**normal 2n** chromosomal complement). Even a small amount of non-cancer tissue (5–10%) will substantially alter the appearance of logR and Ballele plots of certain changes, such as LOH and homozygously deleted (HD) regions (discussed in detail below). Since the proprietary algorithms have been developed and optimized for use with cell lines they perform poorly for tumour biopsies contaminated with stromal tissue.

Several problems arise when attempting to provide a detailed genome-wide profile, particularly in tumour biopsies, all of which can be related to the ability to interpret the critical data captured within the Ballele and logR plots, whether manually, or by some algorithm. Here we describe a simple application which simulates all possible DNA copy number changes in terms of Ballele and logR plots. We believe this tool, Simulated DNA Copy Number (SiDCoN), will be helpful for interpreting complex regions of change, as well as for training researchers to accurately score whole-genome profiles in the presence of significant stromal contamination. Furthermore SiDCoN allows the user to estimate the level of stromal contamination within a tumour sample.

## Results and Discussion

**Figure 1:**
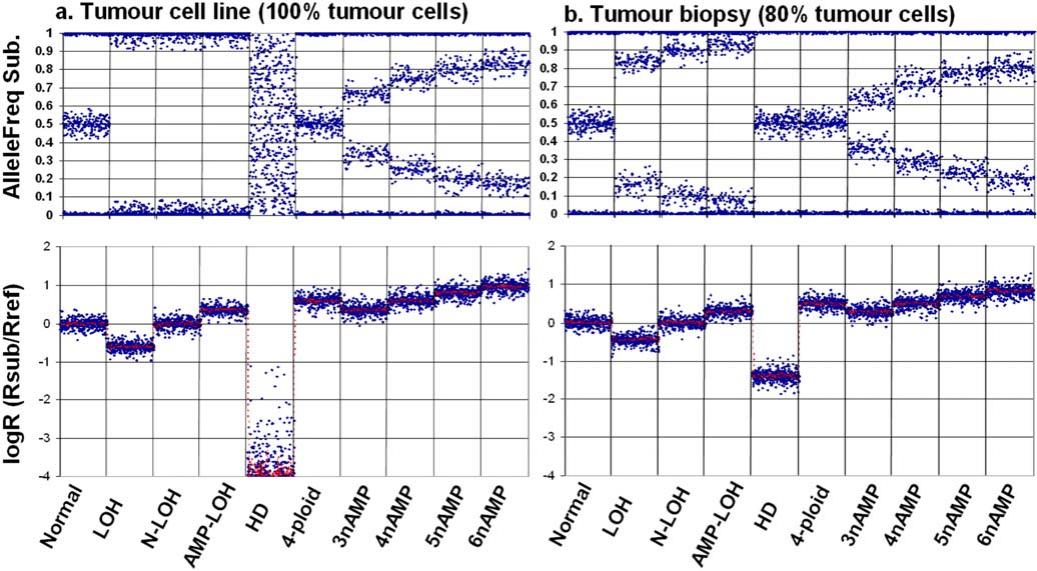
A comparison of DNA copy number genotypes showing Ballele (top) and logR (bottom) simulated data for a) tumour cell line showing 100% changes in each case, and b) tumour biopsy with 80% tumour and 20% stromal (normal) DNA. In each case all common DNA copy number changes are represented (separated by vertical lines) and discussed sequentially within the text.

SiDCoN was used to generate simulated data for all common DNA copy number changes, assuming either no stromal component to the sample, such as seen for a tumour cell line (Figure 1a), or assuming a 20% stromal component, as might be expected for an average tumour biopsy (Figure 1b). The following sections describe the defining features of each DNA copy number variation presented in Figure 1, highlighting the effect the presence of stroma has on the profiles.

### Observable DNA Copy Number Genotypes

#### Normal [2n, AB]

Since stroma is assumed to be normal 2n chromosomal complement, Ballele and logR plots are identical for tumour cell lines (Figure 1a) and tumour biopsies (Figure 1b). The B allele frequencies for each SNP are either close to zero (no B allele) or 1 (2 B alleles) indicating uninformativeness, or close to 0.5 indicating a heterozygous signal with equal proportions of both alleles. Deviations from 0.5 are the result of “random” fluctuations in the experimental system. Across a chromosomal region (multiple SNPs) the relative B-allele intensity sits around 0.5 and the logR is expected to be close to zero; equal proportions of sample and reference DNA resulting in a ratio of 1 and a log2 value of zero. When scoring a region as normal 2n, with a B-allele at 0.5, it is important that the logR be at zero since there are several alterations which have a normal B allele (see HD and 4n normal below). This is one of the major benefits of using a matched normal sample as a reference, rather than the Illumina reference cluster, as described by Peiffer and coworkers [4].

#### Loss of Heterozygosity (LOH) [1n, A or B]

**Figure 2:**
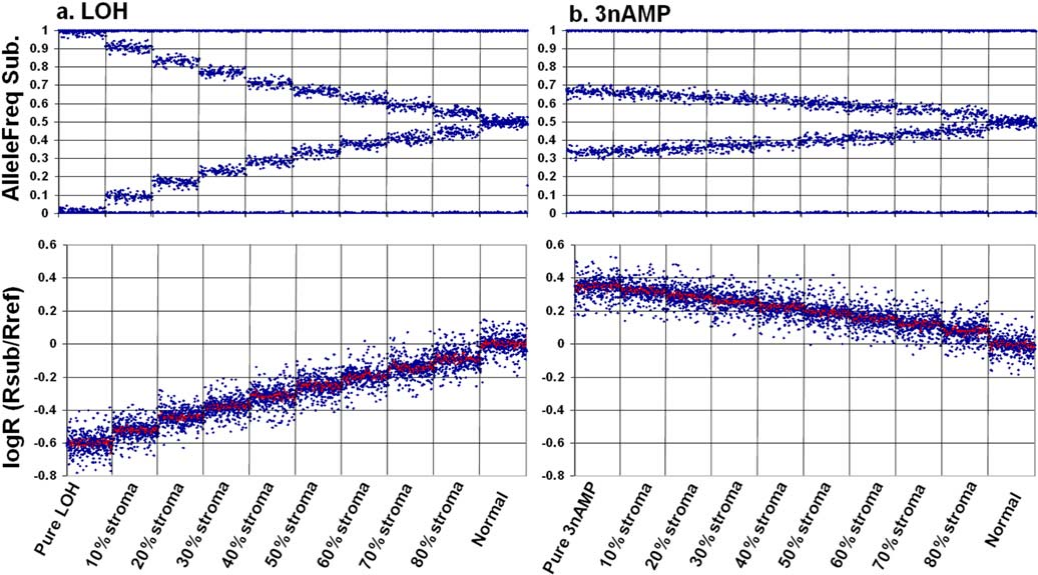
Simulated Ballele (top) and logR (bottom) plots showing serial dilution of the a) loss of heterozygousity, and b) amplification (3n) tumour genotypes in the presence of increasing levels of stromal (normal). In each case stromal levels of 0% (pure tumour) to 80% are represented in steps of 10% (separated by vertical lines), along with the normal 2n genotype.

LOH is one of the most common DNA copy number changes seen in cancer and its occurrence can help localize the position of a tumour suppressor gene. As can be seen from Figure 1 the presence of stromal tissue changes the appearance of the Ballele plot. When the sample contains 100% tumour cells, all with LOH in the region of study, the Ballele plot consists of values either close to zero or close to 1, since there is only one allele present in the sample (Figure 1a). We can label this as pure LOH. However, when stromal tissue is present the allele B frequency for each polymorphic SNP shifts towards 0.5 (Figure 1b). The extent of the shift can be directly proportional to the level of stroma and this phenomenon is described in detail under “Estimating Stromal Contamination” (Figure 2a). Pure LOH results in an expected logR value of minus 0.54 (Illumina Beadstudio manual) as shown in Figure 1a, and, as can be seen in Figure 2a, the presence of stroma makes this signal weaker (closer to zero) in a proportional manner.

#### Copy Neutral LOH (N-LOH) [2n, AA or BB]

N-LOH, or uniparental disomy, is actually the result of two events, first LOH occurs, removing one copy of the target chromosomal region and then this region is duplicated. Alternatively amplification followed by loss of the non-amplified chromosome can result in N-LOH. The resulting genotype is a normal (2n) DNA complement which consists of the same chromosomal version: *AA* or *BB* instead of the *AB* seen in a normal heterozygous region. One of the advantages of using a SNP platform to document DNA copy number changes in tumours is that, unlike karyotype-based CGH methods, N-LOH can be distinguished from normal 2n [1]. With standard LOH methods N-LOH would be seen as LOH, thus specific characterization of N-LOH events is relatively new. N-LOH is surprisingly common in a variety of tumours [1]. As with pure LOH, pure N-LOH results in a Ballele plot that consists of values close to zero or 1, since all the SNPs are homozygous (Figure 1a). When stromal tissue is present the Ballele plot does shift towards 0.5 as seen with LOH, however the degree of this shift is different, but predictable (Figure 1b). The N-LOH genotype results in a logR value that is close to zero (the same as normal 2n tissue) in either the presence or absence of stroma (Figure 1).

#### Amplified LOH (AMP-LOH) [3n, AAA or BBB]

The formation of AMP-LOH is similar to that of N-LOH, except that the chromosomal region resulting from LOH is duplicated more than once. Figure 1 only depicts AAA amplification (3n), although it is possible to use SiDCoN to simulate as many n as required. As with both LOH and N-LOH, pure AMP-LOH will present a B-allele plot consisting of 0 or 1 values (with the system variation) irrespective of how many times the region has been duplicated, and the logR value will approximate that of 3n amplification (∼0.34 – Illumina Beadstudio manual) if there are 3 versions of the target chromosomal region, *AAA* (Figure 1a). When stromal tissue is present the Ballele plot shifts towards 0.5, and again, the degree of shift relates to the amount of normal tissue present (Figure 1b). The level of stromal contamination also controls the strength of the gain observed in the logR. Given the small shift (0.34) expected for pure 3n genotypes, increasing levels of stroma make the logR signal closer and closer to zero. With the degree of variation within the logR signal it is often difficult to distinguish AMP-LOH from N-LOH in the presence of more than 60% stroma.

#### Homozygous Deletion [0n, 0]

The most apparent change associated with a pure homozygous deletion (HD) in cell lines is a very low logR score, below −2 and often dipping below −4. In the presence of stroma, however, the magnitude of the change can be substantially smaller, depending upon the amount of non-cancer tissue present. In the case of complete HD within a cell line, where essentially no DNA remains for the chromosomal region in question, there is not enough signal for an accurate determination of the allele B frequency of each SNP, resulting in essentially random numbers for the Ballele plot (Figure 1a). In the presence of even a trace of non-cancer cells there is sufficient material to gain an accurate allele B frequency, however, since this arises solely from non-cancer tissue the Ballele plot appears as normal 2n. Therefore, unlike pure HD, HD in the presence of stroma can only be distinguished from normal 2n by the drop in logR (Figure 1b). It should be noted that in tumours HD is often mixed with LOH, as discussed below.

When scoring HD on the X chromosome of male subjects, it should be remembered that the normal state (one X chromosome) has a 1 or 0 Ballele pattern. Thus the loss of the remaining allele is only evident by the drop in logR value. The difference between HD in the presence or absence of non-cancer cells is only evidenced by the degree of logR drop (data not shown).

#### 4n Balanced Amplification [4n, AABB]

When the normal chromosomal complement (2n) is duplicated within the target region the logR plot is representative of 4n, while the Ballele plot shows a normal 0.5 signal (Figure 1). In the case where there is a mixture of normal stroma and target-specific tetraploid cells, the strength of the logR signal increase is reduced, while the B allele is unchanged (Figure 1b vs Figure 1a). If a proportion of the tumour sample is tetraploid (4n total chromosome complement), which often happens in a number of cancer types, this is not detectable by current SNP-aCGH analyses, due to the necessity to normalize DNA content between sample and reference.

#### Simple Amplification (3nAMP) [3n, AAB or ABB]

In combination with LOH, regions of genomic amplification are considered hallmarks of the tumour genotype. While LOH often denotes the presence of a tumour suppressor, amplification is indicative of gene(s) that have an oncogenic effect. Given the small shift of 0.34 expected for pure 3n amplified genotypes (Figure 1a), the presence of a moderate proportion of stroma shifts the logR signal close to zero (Figure 1b), such that it is often difficult to distinguish 3n from 2n using logR alone (Figure 2b). In pure 3nAMP the Ballele plot has a characteristic split, with clusterings at 0.66 and 0.34 amongst polymorphic SNPs (along with nonpolymorphic SNPs which cluster at either 1 or 0), indicating that either 2/3 or 1/3 of the alleles are B at individual SNPs (Figure 1a). Consistent with the other changes described above, the presence of stroma shifts the Ballele signal back towards 0.5. The degree of shift is proportional to the percentage of cells with a normal genotype, as shown in Figure 2b. Note the distinctions in Ballele plot between 3nAMP (*AAB*) and AMP-LOH (*AAA*) making it possible to clearly distinguish these two genotypes, even in the presence of stroma (Figure 1).

#### Complex Amplification [e.g. 4n, AAAB]

Higher levels of amplification are reasonably common in tumour samples, e.g. around oncogenes such as ERBB2 and MYC. They differ from 3nAMP in the degree of shift towards 1 in the Ballele plot and increasing logR values, as the ratio of amplified to normal alleles increases in relation to the total number of DNA copies. As the number of copies increases, the possible number of genotype combinations also grows, although generally one allele is chosen to be overrepresented. Figure 1 illustrates 4nAMP (*AAAB*), 5nAMP (*AAAAB*) and 6nAMP (*AAAAB*). Note the clear distinction in B allele plot for 4nAMP (*AAAB*) and 4n-ploidy (*AABB*) in Figure 1. In the presence of stroma it becomes very difficult to unambiguously distinguish 3nAMP from 4nAMP unless the degree of stromal contamination is known (data not shown). Using SiDCoN one can demonstrate that the allelic mixtures for higher levels of amplification (>6n) are difficult to distinguish, especially in the presence of stroma (data now shown).

### Esimating Stromal Contamination in Tumour Samples

Cancer researchers often only have access to small biopsies of tumours, from which accurate estimates of stromal contamination can be difficult. Visual estimates based on the histologic appearance of a neighbouring tissue section can be used as a guide, but there is no guarantee that the section is representative. If the biopsy is small, halving it for pathology considerably reduces yield (often disproportionately) and the tumour content estimate is still only a guide. For both expression and DNA copy-number profiling the presence of large amounts of stromal tissue will reduce the number of differences observed; introducing a type 2 error.

As can be seen in Figure 2, the amount of non-cancer tissue present within a tumour alters both the B allele and logR signals for several copy number changes. We propose that at the site of any of the simple copy number changes seen in tumours (LOH, N-LOH & 3nAMP) the degree of shift can be used to estimate the percentage of cells that have a normal 2n genotype. Using SiDCoN we have been able to assess the tumour/normal DNA mixing experiment presented in Figure 5 (plates A-E) of Peiffer et al [4]. By simulating various ratios of normal and LOH genotypes it is possible to estimate the tumour DNA content in each of the 75%, 50% and 25% mixtures generated by Peifer and coworkers as approximately 71%, 45% and 22% respectively, based on the Ballele plots as presented. In addition, the tumour content in figure 5, plate F of Peiffer et al [4] can also be estimated from the LOH stretch present across the later half of chromosome 13. We estimate that the 0.67 Ballele split corresponds to 52% tumour (or ∼50% from the serial dilution presented in Figure 2a), rather than 67% as described in their legend to figure 5 [4]. Peiffer and coworkers have not accounted for the fact that the LOH genotype contains only one chromosome (*A*), while the normal 2n (stromal) component contains both *A* and *B* chromosomes. Thus, the calculation actually is 2 – (1/0.67), rather than 0.67/1, as they assume. We thought it important to highlight this point since this is the only other published example of applying Ballele and logR plots to estimating stromal contamination level.

**Figure 3:**
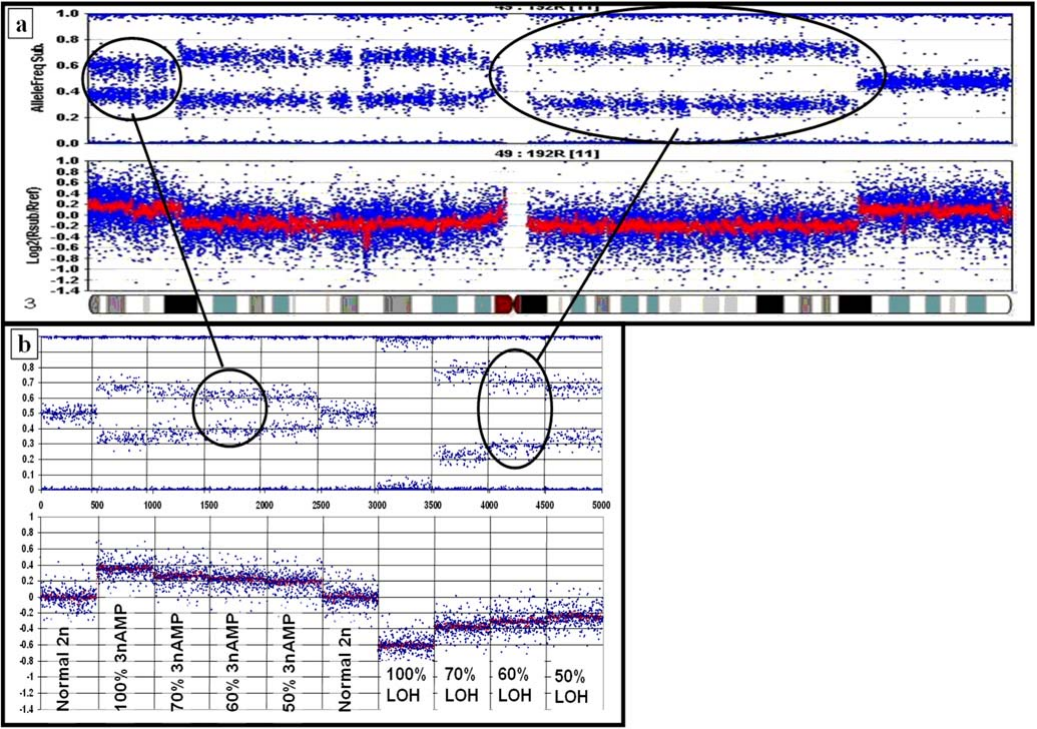
Estimating AMP & LOH levels in a) real data using b) SiDCoN. Comparison to simulated data indicates that ∼60% of cells are AMP or LOH for the indicated regions (thus 40% of cells are normal 2n in each case). These data suggest that this tumour biopsy contains 40% stroma, although more copy number changes across the genome are needed to confirm this.

Figure 3 demonstrates the use of SiDCoN (Figure 3b) to determine the region-specific level of involvement in a specific DNA sample (Figure 3a). In this example, the sample shows 40% normal cells for both the indicated 3nAMP (p arm) and LOH (q arm), based on the visual similarity of these profiles to that of the actual data; particularly the Ballele plot. Using this visual approach, the level of cellular involvement can be estimated for each DNA copy number change present in a sample.

Furthermore, when multiple DNA copy number changes are present in a sample it is possible to estimate the degree of stromal contamination present in a given tumour biopsy, with the aid of SiDCoN. There are two major complications to this procedure:

#### 1) Multiple regions of DNA copy number change are required

At any given region of the genome a tumour might be heterogeneous for a DNA copy number change, such that a proportion of the tumour cells show a change and the remainder are normal or show a different DNA copy number change. Thus, estimating the stromal contamination rate from a single DNA copy number change may be inaccurate. In addition, across a given chromosome, the pattern of DNA copy number change is likely to vary. One must be certain to choose consistent regions of change. For example across a given chromosome arm the level of LOH may vary. The region of LOH which is closest to the Ballele plot expected for pure LOH (Figure 1a) is most likely to give an accurate estimation of the percentage of stroma present. Note that the X chromosome should be excluded from this estimation in samples of male origin.

#### 2) Scoring ambiguity

As discussed above, at higher levels of stromal contamination it becomes difficult to clearly determine the specific tumour genotype in a given region. Estimating the level of stroma from LOH is the most accurate, simply because the Ballele pattern is the most different from normal. Experimentally we find we can score LOH down to 30% of the sample (Figure 2a), however this is dependent on the ability to discriminate LOH from N-LOH on the logR plot, and thus a straight baseline is required.

Taking these factors into consideration, when a primary tumour sample exhibits a number of unambiguous LOH changes across the genome, we can use the Ballele plot level of these to estimate the level of contaminating normal stromal material, as shown from the set of simulations provided in Figure 2 where LOH and AMP changes are provided in 10% steps. Practically, we limit our serial steps to 5% when using SiDCoN in this manner, although either 5 or 10% steps provide a good estimate, depending on the number of scorable changes per sample (down to 30% as described above).

While the amount of stromal contamination does effect how much the logR mean value does shift towards zero, it is not as accurate as using the Allele B frequency for estimating the stromal contamination level.

### Investigating region-specific DNA copy number heterogeneity in tumour samples

**Figure 4:**
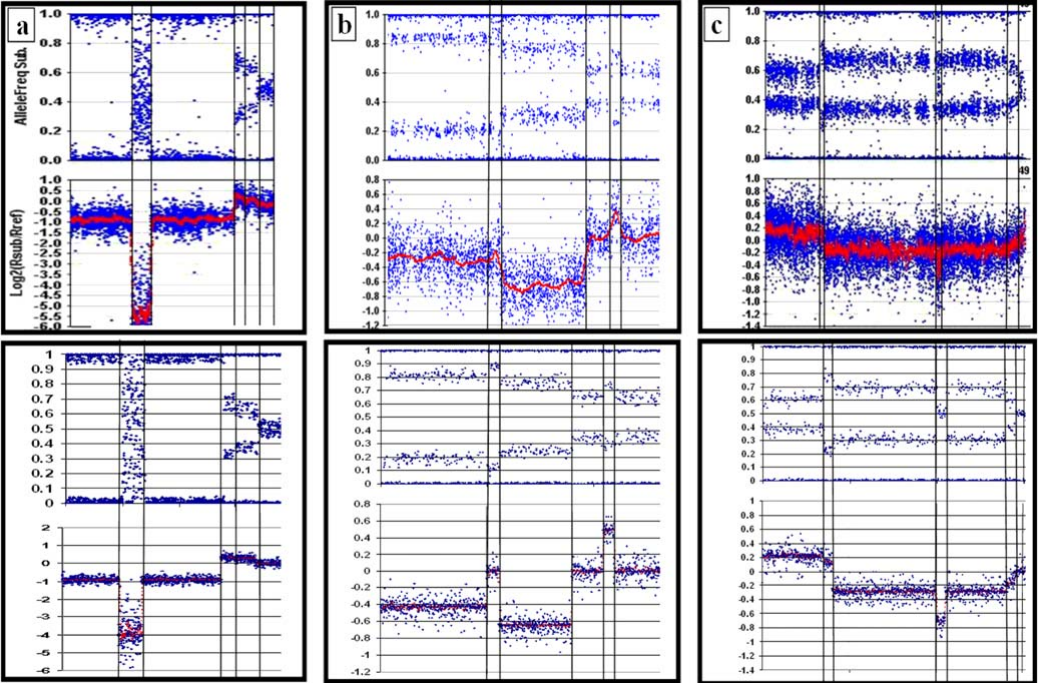
Some examples of observed vs simulated chromosomal excerpts showing mixed populations of DNA copy number changes (top) and the manually adjusted simulations of these changes (bottom). a) a melanoma cell line with changes including HD and mix of LOH & HD. b) an EAC tumour biopsy profile which includes LOH, N-LOH and a mix of HD and LOH. The simulator is particularly useful for explaining LOH/HD combinations in the presence of stroma as seen here. c) another EAC tumour biopsy with changes on a higher background of stroma/normal cells. Manually adjusting the simulator is useful for determining the level of tumour cell involvement in each change.

Figure 4 demonstrates the application of SiDCoN to interpreting the level of DNA copy number change in the presence of stroma (4b and 4c), and estimating the mixture levels of multiple copy number changes (4a and 4b). At the beginning of the melanoma cell line chromosome excerpt depicted in Figure 4a (top) is a region consisting of a combination of LOH and HD cells. Using the simulator (4a bottom) it can be estimated from the logR values that this is the result of 70% of the cell line population being LOH for this region, while the remaining 30% are homozygously deleted. This is consistent with the fact that within this region of 70% LOH/30% HD, is a 100% HD region which contains a known melanoma tumour suppressor gene (CDKN2A). Similarly, in Figure 4b (top) the central section of the chromosomal excerpt from an oesophageal adenocarcinoma tumour biopsy shows a region with a mixture of LOH and HD tumour cells, however in this case normal 2n (stromal) cell population is also present. Note that, at the beginning of this excerpt there is a region of LOH alone, in the presence of a normal 2n population. Using the simulator in the manner demonstrated in Figure 3 we estimate that in each case the normal 2n population is ∼25% and that the 75% LOH region moves to a mixture of 50% LOH and 25% HD within the central region of the section shown (Figure 4b). By looking at LOH regions across all autosomes for this sample, we confirmed that the stromal contamination rate within the biopsy sample is ∼25% (data not shown) suggesting that all tumour cells are involved in the changes described.

As the application of SNP-aCGH to investigate DNA copy number changes becomes more popular, investigators will want to extract as much information as possible from these data. We present here a simple simulation-based method to investigate complex DNA copy number changes to a level beyond current analytical methods. While the application of our method is manual, in relation to users adjusting the simulated parameters to obtain a visual match for individual data, SiDCoN provides researchers with the flexibility to assess a wide variety of SNP-aCGH data interpretations. We have demonstrated how this approach can be used to estimate the stromal contamination rate within tumour biopsies, and to describe mixed DNA copy number populations, in the presence or absence of normal 2n cell populations. There is also the potential to implement the basic principles of our application into next-generation autoscoring programs which may save researchers considerable time.

## Methods

### Test samples

DNA was extracted from melanoma cell lines and oesophageal adenocarcinoma biopsies using Qiagen nucleic acid column purification technology as set out in the manufacturer's instructions (Qiagen, Hilden, Germany). The resulting genomic DNA was quantitated using a Nanodrop and 750ug applied to Infinium II Whole Genome Genotyping HumanHap300 Beadarray chips as per manufacturer's instructions (Illumina, San Diego, CA, USA). The chip image data was then processed through Beadstation and Beadstudio 2 software applications to generate B-allele and logR plots. In the case of melanoma cell lines, the Illumina reference pool was used as a reference. For the adenocarcinoma biopsies a normal squamous oesophageal biopsy (from one of the cancer patients) was used as a reference. The normal squamous tissue was compared to the Illumina reference pool to look for any anomalous regions. This highlighted a small region of change on 6q which was excluded from the analyses of the oesophageal tumour biopsies. We found, as reported by Peiffer and co-workers [4], that using a local normal tissue in this manner gave a much cleaner baseline signal compared to using the Illumina pooled reference.

### Using SiDCoN

**Figure 5:**
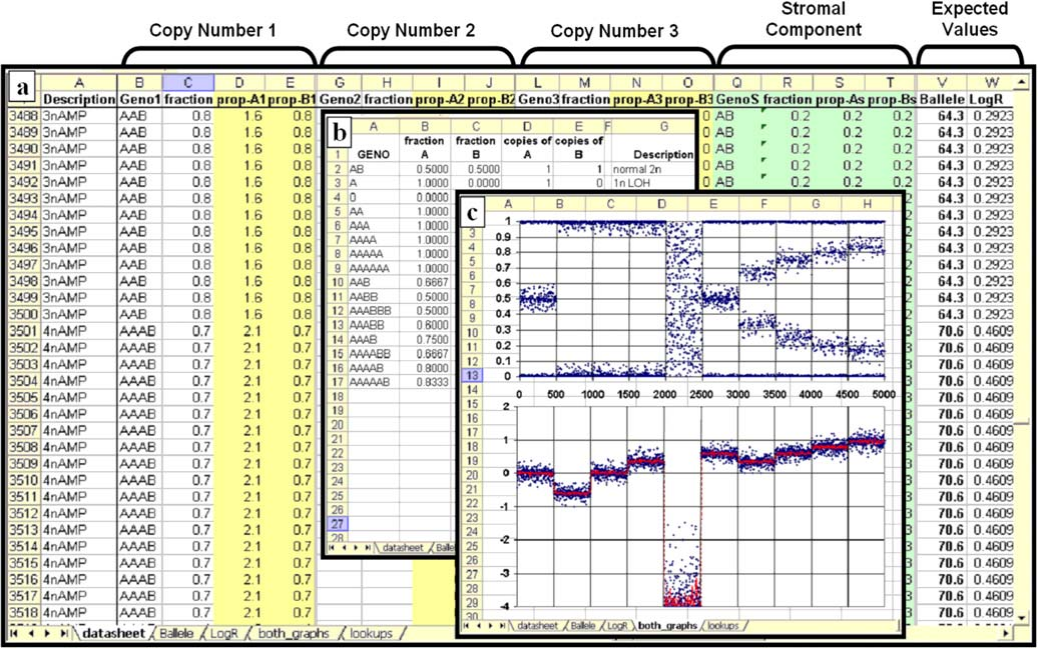
Screen grabs from SiDCoN showing a) the main “datasheet” interface with space for three DNA copy number genotypes and a stromal component for each SNP, b) the lookup sheet containing information needed for calculations dependent on the copy number genotypes entered and c) the Ballele and logR output, implementing randomised values to visually simulate the look of actual data.

SiDCoN (Supplement S1) is an Microsoft EXCEL spreadsheet application that allows users to enter up to 3 copy number genotypes, along with a stromal proportion, for up to 5000 observable datapoints (SNPs). Supplement S2 is a help file outlining its usage. Generally, we envisage its use will be restricted to estimating one genotype and stromal contamination, or a mixture of 2 copy number variants; however we wanted the tool to be as flexible as possible. Each copy number genotype is entered into the main “datasheet”, along with the proportion of cells (0–1) with this type (Figure 5a). If the total of the 3 genotypes for a SNP is less than 1, the remaining portion is assumed to be stroma (normal 2n, AB). The relative proportion each entered DNA copy number genotype makes towards the overall pattern is determined using predetermined values from the “lookup” sheet, shown in Figure 5b, and the entered fractions. From these calculations the expected Ballele and logR values are determined for each SNP (columns V and W on the right hand side of Figure 5a). A randomization algorithm is then applied to each datapoint, using the EXCEL “RAND()” function, to allow the simulated data plots to appear visually similar to real data. The resulting Ballele and logR plots are then presented as EXCEL graphs on separate sheets (not shown), or combined on the “both graphs” sheet (Figure 5c). The results (Figure 1) provide a means to generate reasonable facsimiles of actual DNA copy number data, enabling the user to determine how best to interpret patterns observed in the real data.

Ballele frequency graphs focus around 3 (normal 2n) or 4 lines which correspond to whether the B allele for each SNP is informative or uninformative with either no copies (0) or all copies (1) of allele B. When the B allele is informative it can either be the predominant SNP allele (>0.5), the minimal SNP allele (<0.5) or in equal proportion with A (0.5). The latter case, results in 3 focus lines (1, 0.5 & 0) (see Figure 1a, normal & 4-ploid). Instances where there is only 1 parental chromosome present result in only 2 lines of focus (0 & 1) (see Figure 1a, LOH, N-LOH and AMP-LOH) while all other instances result in 4 lines of focus. Since all SNPs in the current assay have only two possible alleles (A & B) this is always the case (even for sex chromosomes), unless there are more than 2 parental chromosomes present. To be consistent with Peiffer and coworkers [4], we represent Ballele as the position of the upper informative line of focus, giving a range of 0.5 to 1. A value of 1 means only one parental chromosome is present such that no SNPs are informative (lines of focus at 0 and 1) as is the case for complete LOH. A value of 0.5 indicates a normal (2n) Ballele appearance with lines of focus at 1, 0 and 0.5, while other values (between 1 and 0.5 indicate there are 4 lines of focus 1, 0, Ballele and 1-Ballele).

SiDCoN generates simulated mean logR values using the formula:
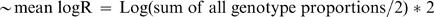
We expected that log2 would yield the correct result, but for some reason log10, in the simulations, provides much closer values to observed logR values. Presumably this is the result of using simulated R values.

## Supporting Information

Supplement S1 Simulated DNA Copy Number (SiDCoN) - a spreadsheet application designed to simulate the appearance of B-allele and logR plots for all known types of tumour DNA copy number changes in the presence or absence of stromal contamination
(4.89 MB XLS)

Supplement S2 Instructions for using SiDCoN
(0.03 MB DOC)
