# Supplementary material for: SiDCoN: A Tool to Aid Scoring of DNA Copy Number Changes in SNP Chip Data
Source: PLoS One. 2007 Oct 31;2(10):e1093. doi: 10.1371/journal.pone.0001093 (PMC2034603; doi:10.1371/journal.pone.0001093)
Supplement: Supplement S2 — Instructions for using SiDCoN (0.03 MB DOC) [file pone.0001093.s002.doc]

**How to use the SiDCoN simulator:**

SiDCoN (Simulated DNA Copy Number) is designed to help confirm manual predictions of DNA copy number assignments using data generated from high density SNP arrays (SNP-aCGH, eg Illumina Infinium II Whole Genome Genotyping HumanHap300 Beadarray chips). For example, Ballele and logR output graphs generated by Illumina BeadStudio 2 or 3 for a particular sample look as though a certain chromosomal region contains 70% heterozygous deletion (LOH), 10% homozygous deletion (HD), and 20% stromal contamination. To be sure of this one can use SiDCoN to simulate whether the expected Ballele and logR from this prediction match the observed graphs. SiDCoN is especially useful for generating expected outcomes from complicated heterogeneous samples, but it can also be used to train personnel to identify particular DNA copy number changes, act as a focal point to discuss contentious regions of change amongst scorers, generate simulated copy number data containing specific genotypes (to test an autoscoring algorithm, for example), and to estimate the fraction of normal (2n) cells within a tumour sample.

Within the Excel file, there are five worksheets:

1. datasheet
2. Ballele
3. logR
4. both_graphs
5. lookups.

Data is entered into the “datasheet” and the results of this input can be seen as Ballele and logR plots on the second and third worksheets, respectively. If you want to view both Ballele and logR plots at the same time, you can see them on the fourth worksheet “both_graphs”. The “lookup” sheet contains the values/constants SiDCoN uses to make genotype specific calculations.

Within “datasheet”, note the following aspects.

*The columns*:

- Avoid typing in the coloured columns and cells as these contain formulae.
- Column A “Description” - a text label for the genotype to be simulated for each “SNP” e.g. “LOH plus HD”, “stroma plus LOH”, “normal”, etc. This column is for your own reference and not used by SiDCoN in any way.
- Column B “Geno1” - the primary genotype for each “SNP” using the appropriate genotype code. The code for each genotype is shown in the “GENO” column of the “lookup” sheet. For example, normal 2n is represented by “AB” and “0” (zero) represents homozygous deletion (HD). Currently SiDCoN is set up to accept all detectable genotypes up to and including 6n, however more complex genotypes can be added to the lookup table when required. To test multiple genotype combinations a second genotype can be entered in Column G “Geno2”, and the other on Column L “Geno3”.
- Column C “fraction” - the required fraction (0-1) of the genotype specified in column B for each “SNP”. For example, to check 95% LOH, enter “A” into Column B (the genotype code for LOH) and 0.95 into Column C for the chosen “SNP”. The remaining 5% (0.05) will be automatically assigned as stroma (normal 2n), assuming “Geno2” and “Geno3” are not used. Similarly, column H is for the proportion of Geno2 and M for the proportion of Geno3.
- The seven columns discussed above are the only “datasheet” columns where data should be entered into.
- Columns D, E, I, J, N and O, coloured yellow - used to calculate the contributing proportions each allele (A or B) makes towards the overall genotype by looking up the appropriate values in the “lookup” sheet. As mention above, do NOT type anything on the coloured cells.
- Columns Q to T, coloured green, - used to automatically calculate the proportion of the stromal contamination (assumed to have “AB” or normal 2n genotype).
- Columns V and W - the expected Ballele (assuming the “SNP” is polymorphic) and logR values, respectively, based on the entered genotype information.
- Columns X to AE - used to plot the data, introducing a random component to simulate the visual appearance of real data. The degree of random spread can be adjusted using cell Y1. The standard/default value is 0.1.

*The rows*:

Each row represents a “SNP”. Currently SiDCoN is set up to simulate 5000 SNPs. More rows can be added by copying from the current calculation cells and adjusting the plot ranges accordingly. Note that this will require additional memory and hamper SiDCoN’s performance.

To generate easily viewable Ballele and logR plots, sets of consecutive 500 row (“SNP”) lots can be assigned to the same genotype (type the first row and fill down). Alternatively the number of rows assigned to each genotype can be manually matched to visually represent the sample being simulated. For example, if a real sample has ~500 SNPs that look as though the first 350 SNPs contain 70% LOH and the remaining 150 appear to be 70% 3nAAA amplification, you can input code “A” and fraction 0.7 in rows 2 to 350 and code “AAA”/fraction 0.7 in rows 351 to 500.

A further option is to use a section of rows to generate a serial dilution for a particular genotype combination. For example, fractions of LOH (code “A”) from 0 to 1 can be entered into concurrent column C cells (eg using a cell above + 0.01 formula) to generate a table of possible LOH/stoma mixtures, which can be used to determine the fraction of LOH present in a particular LOH region. In this case the observed (real data) polymorphic Ballele fraction is compared to the expect Ballele values (“datasheet” column V) for each dilution.

Once you finish entering your data, you can immediately check the Ballele and logR plots on their respective, or the “both_graphs”, worksheets. These graphs can be copied and pasted (as picture only) into Powerpoint or other applications for independent viewing.

Note: For a total HD, that is when you put “0” into Column B “Geno1” and “1” in Column C “fraction”, your simulated logR is -4 (minus 4), while the observed logR can be lower than this. In addition, for this genotype the expected “Ballele” value will generate a division by zero error, which SiDCoN uses to identify this as a complete HD.
